# Frontal Theta Activity Supports Detecting Mismatched Information in Visual Working Memory

**DOI:** 10.3389/fpsyg.2017.01821

**Published:** 2017-10-17

**Authors:** Tengfei Liang, Zhonghua Hu, Qiang Liu

**Affiliations:** Research Center of Brain and Cognitive Neuroscience, Liaoning Normal University, Dalian, China

**Keywords:** mismatch, frontal theta activity, visual working memory, control processing, delayed matching task

## Abstract

During the comparison stage of visual working memory (VWM) processing, detecting the mismatch between the external sensory input and internal representations is a crucial cognitive ability for human, but the neural mechanism behind it remains largely unclear. The present study investigated the role of frontal theta power in detecting the mismatched information in VWM in a delayed matching task. A control task required to compare two simultaneously presented visual figures was also designed as a contrast to exclude the possibility that frontal theta activity just reflecting the non-memory-related behavioral conflicts. To better characterize the control mechanisms shaped by the frontal theta oscillation in human VWM, colored shapes were adopted as materials while both the task-relevant shape feature and task-irrelevant color feature could be mismatched. We found that the response times of participants were significantly delayed under the relevant- and irrelevant-mismatch conditions in both tasks and the conjunction-mismatch condition in delayed matching task. While our EEG data showed that increased frontal theta power was only observed under the relevant- and conjunction-mismatch conditions in the delayed matching task, but not the control task. These findings suggest that the frontal distributed theta activity observed here reflects the detection of mismatched information during the comparison stage of VWM, rather than the response-related conflicts. Furthermore, it is consistent with the proposal that theta-band oscillation can act as a control mechanism in working memory function so that the target-mismatched information in VWM could be successfully tracked. We also propose a possible processing structure to explain the neural dynamics underlying the mismatch detection process in VWM.

## Introduction

As a pivotal cognitive system, working memory allows for transiently storing and utilizing of information (Baddeley, 1992, 2001). As a function of this cognitive system, comparing the representations stored in visual working memory (VWM) with the perceptual input is of considerable importance to both high-level and low-level visual processing (Luck, 2008). This process requires updating relevant memory representations and adjusting the current cognitive operation, which consequently promotes appropriate behavior in accordance with the external environment (Hollingworth et al., 2008; Richard et al., 2008). Previous studies have revealed that the detection of the mismatched item during the comparison stage of VWM first evokes a bottom-up attention capture (Eimer and Mazza, 2005; Hyun et al., 2009). Neuroimaging studies further indicated that detecting mismatch in the context of VWM can also activate the frontal region (Pessoa and Ungerleider, 2004; Zhang et al., 2008), mainly including the right dorsolateral prefrontal cortex, indicating the working memory related control processing. However, little is known about whether there are any neural oscillations that corresponding to this control processing.

Oscillatory activities of the brain carry important information about cognitive processing, but cannot be detected by using traditional time-domain methods (Makeig et al., 2004). In these neural activities, oscillation in the theta-band (4–8 Hz) over the frontal regions was thought to be responsible for the control process of working memory functions (Sauseng et al., 2010). For example, increased theta power has been observed during the encoding and retention stage of working memory processing (Raghavachari et al., 2001; Jensen and Tesche, 2002; Meltzer et al., 2008). These oscillatory activities have also been reported to be involved in memory retrieval (Karrasch et al., 2004; Sauseng et al., 2004) and the maintenance of temporal order information (Hsieh et al., 2011). Given the functional significance of frontal theta oscillation to working memory processing (Hsieh and Ranganath, 2013), it seems that they may also be involved in detecting the mismatched item during the comparison stage of VWM. What is noteworthy is that this process could also induce competitive conflicts on behavioral responses (Eriksen and O’Hara, 1982; Pan and Eriksen, 1993). The monitoring of the response-related conflicts was suggested to require the involvement of the anterior cingulate cortex (Barch et al., 2001; Van Veen and Carter, 2002) and the oscillatory dynamics in the theta-band of frontal region (Hanslmayr et al., 2008; Cavanagh et al., 2009). The divergence of the above views about the functional significance of frontal theta oscillation thus rises a question. If these oscillatory activities could be observed in the mismatch detection process of VWM, are they supporting the VWM-related processing, or related to the general behavioral conflicts? The answer to these questions will greatly improve our understanding about the relationship between frontal theta oscillation and the mismatch detection process in VWM.

To this end, a visual delayed-matching task was used to investigate whether the frontal theta activity supports detecting the mismatched item during the comparison stage of VWM. Taking into account the fact that iconic memory trace of the first item could last about 250 ms (Gegenfurtner and Sperling, 1993), the blank interval of this task was extended to 1000 ms to ensure that the first item was stored in VWM. Such blank interval was also widely used in previous visual work memory studies (Luck and Vogel, 1997; Jiang et al., 2000). Although by virtue of this task, the mismatch detection-related oscillatory activities could directly be obtained, however, as described above, the mismatch between memory representation and incoming sensory input would inevitably induce response-related conflicts. Thus, this paradigm cannot establish an exclusive relationship between the observed oscillatory activities and the mismatch detection process in VWM. In order to solve this dilemma, a control task was designed, which required detecting the mismatch between two visual figures presented simultaneously. Compared with the delayed-matching task, when participants do the mismatch detection process in the control task, only the non-memory-related behavior conflicts would be elicited. If the observed frontal theta activities were related to the general behavior conflicts, it can be predicted that in both tasks, these oscillatory activities should have the same changing trend as the external behavioral responses (e.g., as expressed in the reaction times). Another question that we were concerned about is whether frontal theta activity is related to the control process of VWM function. For this purpose, colored shapes were adopted as materials while both the task relevant shape feature and irrelevant color feature could be mismatched. We predict that frontal theta power could only be elicited by the task-relevant feature mismatch, which reflects the active involvement of VWM-related control processing.

## Materials and Methods

### Participants

Twenty (14 males) healthy undergraduates from Liaoning Normal University were tested. All participants reported normal or corrected-to-normal vision without known cognitive or neurological impairments, and all of them are right-handed. Participants received a small amount of money for participation. This study was carried out in accordance with the Declaration of Helsinki, and written informed consent was secured from all participants. The protocol was approved by the institutional ethics committee of the Liaoning Normal University.

### Stimuli

**FIGURE 1:**
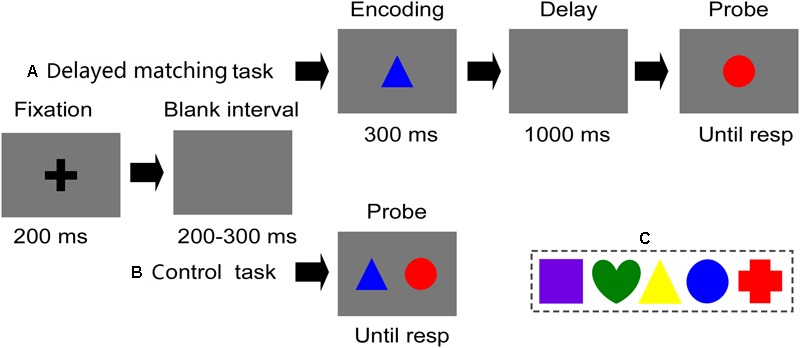
Stimuli and experimental procedure of both tasks used in crrent study. **(A)** Illustrates the time course of a trial in the delayed matching task. **(B)** Illustrates the time course of a trial in the control task. In these trials, two figures were mismatched in their shape (relevant) and color (irrelevant) features. **(C)** Five distinct shapes and colors used in current study.

Stimuli were presented at a viewing distance of 70 cm on a 17-inch CRT monitor (1024 pixels × 768 pixels, 85 Hz refresh-rate) with a gray background. Each image was a colored shape (2.38° × 2.38° of visual angle), which consisted of one of five shapes (crisscross, round, triangle, square, and heart) and colors: green (0,255,0), red (255,0,0), yellow (255,255,0), blue (0,0,255), and violet (255,0,255). All of these features were selected randomly. All of these features were selected randomly (**Figure 1C**). For the control task, two stimuli were presented simultaneously in two fixed locations horizontally (and symmetrically) on either side of the fixation point. For the visual delayed-matching task, two figures were presented sequentially in the center of the screen.

### Experimental Procedures

**Figures 1A,B** show a representative sequence of trials and the detailed timing of one trial for the visual delayed-matching task and the control task. In each trial, stimulus was presented as follows: Firstly, trials began with a fixation for 200 ms followed by a 200–300 ms blank interval. For the control task, two stimuli were presented simultaneously, which remained on the screen until a response was initiated. While for the visual delayed matching task, one memory item (S1) was displayed for 300 ms, followed by a 1000 ms blank interval. Then, the test item (S2) was presented, which remained on the screen until a response was initiated. In visual delayed matching task, participants were required to remember the figure’s shape while ignoring its color, and judge whether the shape of the probe figure was matched as that of the stored figure. While in control task, participants were instructed to detect the mismatch of the target shape features between two simultaneously presented figures, while ignoring their color features. Participants were seated in a chair in a sound-shielded room, and were informed to press “F” on the keyboard for mismatch trials and “J” for match trials as quickly and accurately as possible. Once the response was initiated, a 1000–1400 ms blank interval would be presented before the next trial.

For both of the delayed matching task and control task, four distinct experimental conditions were employed: (1) two figures were complete matched in their shape and color features (Match); (2) two figures were mismatched in their relevant shape features, while their irrelevant color features were matched (Relevant mismatch); (3) two figures were mismatched in their irrelevant color features, while their relevant shape features were matched (Irrelevant-mismatch); (4) for the two figures, both of their shape features and their color features were completely mismatched (Conjunction-mismatch). The whole experiment was divided into two sessions with the order being counterbalanced among participants. Each session corresponds to a task and consisted of eight blocks (50 trials in each block), with a total of 800 trials and duration of about 1.5 h. There were 100 trials for each experimental condition of both tasks, with the same number of four distinct experimental conditions randomly intermixed in each block.

### EEG Data Recording

Continuous electroencephalograph (EEG) signals were recorded by a 64-channel amplifier (500 Hz sampling rate, ANT Neuro EEGO Inc., Germany) via a elastic cap, containing 64 unshielded and sintered Ag-AgCl electrodes, with all of the electrodes layout according to the International 10–20 electrode system. Data were re-referenced offline to the average of the mastoids. For monitoring ocular movements and eye blinks, electroculogram (EOG) recordings were taken from four electrodes placed lateral to each eye and above and below the right eye. Electrode impedance was always kept below 10 kΩ.

### EEG Data Analysis

For the time-frequency analysis, EEG data were imported and processed by EEGLAB (Makeig et al., 2004) and Letswave (Mouraux and Iannetti, 2008). Continuous EEG data were band pass filtered between 1 and 30 Hz. EEG epochs were extracted with a time window of 2000 ms (-500 ms pre-stimulus and 1500 ms post-stimulus) for independent component decomposition. Then, trials contaminated by gross movements were removed manually. Meanwhile, epochs corresponding to missing trials (trials without responses) or error in both tasks were excluded from analysis (for all the datasets, the ratio between the rejected trials number and all trials number was 17% averagely in each experimental condition of both tasks). EOG artifacts were corrected by adopting an independent component analysis (ICA) algorithm (Makeig et al., 2004). In all datasets (for each dataset, 62 components were extracted), 2.2 components were removed averagely (range, 1–4) per participant. EEG epochs were then re-segmented into 1800-ms epochs (-500 ms pre-stimulus and 1300 ms post-stimulus, such long epoch was in order to overcome the edge effects) for time-frequency analysis. Baseline correction was performed using the pre-stimulus interval (pre-stimulus -500 ms to 0 ms).

The time-frequency representation (TFR) of single-trial EEG epoch was obtained through performing the continuous Morlet wavelet transform (CWT) (Mouraux and Iannetti, 2008) to characterize the amplitude of neural oscillation as a function of time and frequency. The parameters of restriction (σ) and central frequency (ω) in CWT were, respectively, stetted as 0.15 and 5, with exploring frequencies ranging from 1 to 30 Hz in steps of 0.3 Hz. Single EEG trials TFR were then averaged to obtain the general TFR, which would help identify stimulus-induced modulations of ongoing neural oscillations. The magnitude of stimulus-induced changes in EEG oscillation was displayed as an either increase or decrease in the oscillatory power relative to the pre-stimulus interval (-300 to -100 ms, such temporal region was used as a baseline interval so as to avoid edge effects when CWT was performed) according to the following formula:

ER%(t,f) = [P(t,f)−R(f)]/R(f)×100

In this formula, a given time-frequency point (t,f) was calculated by P(t,f) = |F(t,f)|^2^. For each estimated frequency f, the averaged signal power within the reference interval (-300 to -100 ms) was calculated by R(f) (Pfurtscheller and Da Silva, 1999; Zhang et al., 2013).

Power modulation of the oscillation activity after the stimuli onset was analyzed by calculating for every time-frequency pixel in the TFR. An exploratory data-driven analysis was adopted to identify the time-frequency regions of interest (TF-ROIs) and their corresponding spatial regions of interest (S-ROIs) which were modulated significantly by factors of mismatch type and task type or their interaction. To do this, each time-frequency point (t, f) of the ER% maps was compared using a point-by-point two-way repeated-measures ANOVA, with task type (delayed matching task vs. control task) and mismatch type (Irrelevant-mismatch, Relevant-mismatch, Match vs. Conjunction-mismatch) as factors, combined with a cluster-based permutation test (non-parametric statistics). This procedure was repeated 5000 times, yielding a data-driven distribution of a time-frequency map of *F*-value and a time-frequency map of *p*-value, representing the interaction between mismatch type and task type. Time-frequency points with a *p*-value ≤ 0.05 were selected for subsequent analyses. To address the problem of multiple comparisons, the significance level (*p*-value) was corrected using a false discovery rate (FDR) procedure (Durka et al., 2004). Besides, to control the false-positive observations (Maris and Oostenveld, 2007), significant TF-ROIs were defined based on the criteria that included time-frequency pixels were significantly different from pre-stimulus intervals at *p* < 0.01 in both tasks. This was achieved used a bootstrapping method (Durka et al., 2004; Makeig et al., 2004).

A two-way repeated-measures analysis of variance (ANOVA) was used to compare the mean power among the four mismatch types of both tasks within each defined TF-ROI at corresponding S-ROI. In all analyses, Greenhouse-Geisser corrections for non-sphericity were applied where appropriate and Bonferroni correction for multiple comparisons was used.

## Results

### Behavioral Results

**FIGURE 2:**
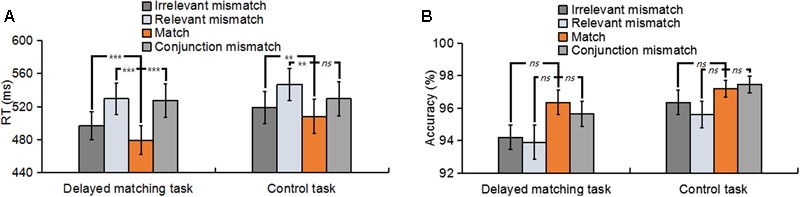
**(A)** The mean reaction times for Irrelevant-mismatch, Relevant-mismatch, Match, and Conjunction-mismatch conditions in both the delayed matching task and the control task. **(B)** The mean accuracies for Irrelevant-mismatch, Relevant-mismatch, Match, and Conjunction-mismatch conditions in both the delayed matching task and the control task. The error bars represent standard errors (SEs), and asterisks mark significant differences between the match condition and other three types of mismatch conditions. ns: non-significant; ^∗^*p* < 0.05; ^∗∗^*p* < 0.01; ^∗∗∗^*p* < 0.001.

Trials with a response time (RT) of less than 200 ms or greater than two standard deviations from the participant’s mean RT were removed. **Figure 2** summarizes the mean RT and accuracy for all mismatch types in both tasks. In terms of the RT, a 2 (task type: delayed matching task vs. control task) × 4 (mismatch type: Irrelevant-mismatch, Relevant-mismatch, Match vs. Conjunction-mismatch) repeated-measures ANOVAs revealed a significant interaction between task type and mismatch type [*F*(3,57) = 5.48, *p* < 0.05, $\eta_{p}^{2}$ = 0.22] and a significant mismatch type main effect [*F*(3,57) = 23.89, *p* < 0.01, $\eta_{p}^{2}$ = 0.56], but no significant task main effect [*F*(1,19) = 0.86, *p* = 0.37]. Analysis of the simple main effects of the interaction between mismatch type and task type showed that in both tasks, RT of Relevant-mismatch was significantly slower than Match (*p* < 0.01) and Irrelevant-mismatch (*p* < 0.01), Irrelevant-mismatch was also significantly slower than Match (*p* < 0.01). The differences of the performance between two tasks was that in delayed matching task, Conjunction-mismatch was significantly slower than Irrelevant-mismatch (*p* < 0.01) and Match (*p* < 0.01), but not Relevant-mismatch (*p* = 1). While in control task, Conjunction-mismatch was significantly faster than Relevant-mismatch (*p* < 0.01), but not Irrelevant-mismatch (*p* = 1) and Match (*p* = 0.149). Those results suggests that in delayed matching task, both of the relevant feature and irrelevant feature were encoded into memory, and for both tasks, whether the mismatched features were relevant or not, they all considerably influenced the performance, thus generated robust mismatch conflicts.

In terms of the accuracy, an identical ANOVA showed a significant task main effect [*F*(1,19) = 5.64, *p* < 0.05, $\eta_{p}^{2}$ = 0.23] and a significant mismatch type main effect [*F*(3,57) = 6.49, *p* < 0.01, $\eta_{p}^{2}$ = 0.26], but no interaction between task type and mismatch type [*F*(3,57) = 0.64, *p* = 0.59]. *Post hoc* analysis of the task main effect showed that accuracy in the control task was better than delayed matching task (*p* < 0.05), indicating more memory retrieval efforts were required in the delayed matching task. For both tasks, *post hoc* analysis showed that there was only a weak significant difference between Conjunction-mismatch and Relevant-mismatch (*p* < 0.05) in control task, with a better behavioral performance in the Conjunction-mismatch condition. The other comparisons among the four mismatch types of the two tasks were all non-significant [all *p* > 0.05]. This accuracy results pattern may arise from the ceiling effect due to the simplicity of both tasks.

### EEG Results

**FIGURE 3:**
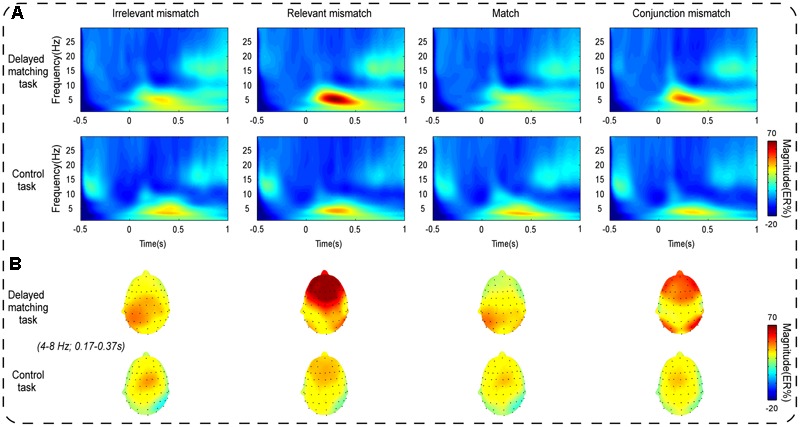
**(A)** Shows the mean time-frequency plots for the Irrelevant-mismatch, Relevant-mismatch, Match, and Conjunction-mismatch conditions in both the delayed matching task and the control task in the S-ROIs of the frontal electrode sites [(Fz + F1 + F2 + F3 + F4 + AF3 + AF4+ FPz)/8]. The modulations of the oscillation power (expressed as ER%) indexes the mismatch detection processing. **(B)** Shows the topography of frontal theta power distributions within the corresponding TF-ROIs (4–8 Hz, 0.17–0.37 s). Time (s) during the comparison period is plotted on the *x*-axis and frequency (Hz) is plotted on the *y*-axis. Time zero indicates the onset of the probe stimulus. Warm/hot colors represent enhanced power.

**FIGURE 4:**
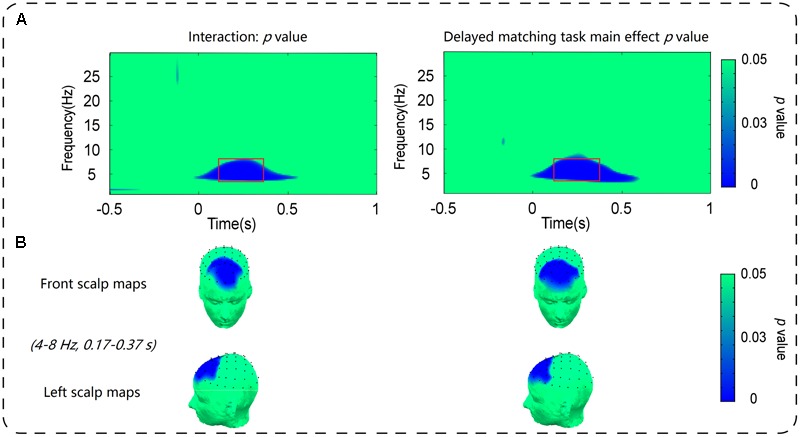
**(A)** Depicts the distribution of the *p*-value of the main effect of mismatch type in delayed matching task in the S-ROIs of the frontal electrode sites [(Fz + F1 + F2 + F3 + F4 + AF3 + AF4+ FPz)/8] and the interaction between mismatch type and task type in the S-ROIs of the frontal electrode sites [(Fz + F1 + F2 + F3 + F4 + AF3 + AF4+ FPz)/8]. The time-frequency pixels displaying significant modulations are outlined in rectangles. **(B)** Shows the topography of *p*-value within the corresponding TF-ROIs (4–8 Hz, 0.17–0.37 s) in the main effect of mismatch type in delayed matching task and the interaction effect between mismatch type and task type. Time (s) during the comparison period is plotted on the *x*-axis and frequency (Hz) is plotted on the *y*-axis. Time zero indicates the onset of the probe stimulus.

**FIGURE 5:**
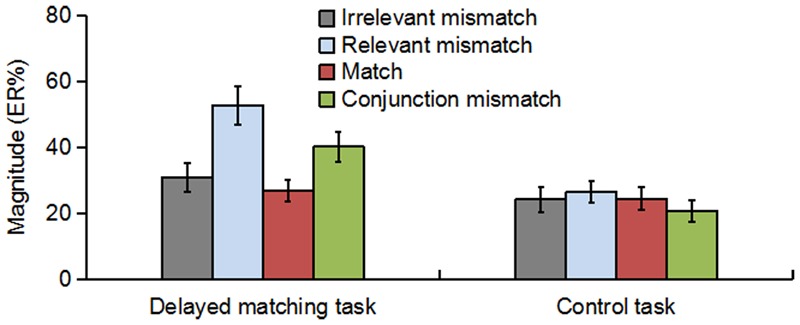
The theta-band oscillation power (expressed as ER%) for Irrelevant-mismatch, Relevant-mismatch, Match, and Conjunction-mismatch conditions in both the delayed matching task and the control task within the TF-ROI (4–8 Hz, 0.17–0.37 s) of the frontal electrode sites [(Fz + F1 + F2 + F3 + F4 + AF3 + AF4+ FPz)/8]. The error bars represent standard errors (SEs).

As shown in **Figure 3**, the oscillatory activities of theta-band showed a clear frontal maximum. Confirming this observation, the exploratory data-driven analysis revealed a significant interaction effect of mismatch type by task type happened mainly in frontal electrode sites (Fz, F1, F2, F3, F4, AF3, AF4, and FPz). A TF-ROI including the theta-band (4–8 Hz, 0.17–0.37 s) that showed the most pronounced interaction-related effect was defined (shown as rectangles in **Figure 4A**). Mean oscillation power within this defined TF-ROI (expressed as ER%) was then entered into a 2 (task type: delayed matching task vs. control task) × 4 (mismatch type: Irrelevant-mismatch, Relevant-mismatch, Match vs. Conjunction-mismatch) repeated-measures ANOVAs (**Figure 5**). This results revealed a significant mismatch type main effect [*F*(3,57) = 9.72, *p* < 0.001, $\eta_{p}^{2}$ = 0.34], a significant task type main effect [*F*(1,19) = 8.55, *p* < 0.01, $\eta_{p}^{2}$ = 0.31] and a significant interaction effect of mismatch type by task type [*F*(3,57) = 11.46, *p* < 0.001, $\eta_{p}^{2}$ = 0.38]. Further analysis of the interaction effect showed that there was a main effect of mismatch type in the delayed matching task [*F*(3,57) = 16.59, *p* < 0.001, $\eta_{p}^{2}$ = 0.47], but not the control task [*F*(3,57) = 1.09, *p* = 0.353]. The main effect of mismatch type in delayed matching task was also confirmed by the data-driven analysis, which showed significant difference in theta-band power of the frontal region (**Figure 4B**) with a similar TF-ROI. *Post hoc* analysis of this main effect showed that frontal theta power (expressed as ER %) was strongest in Relevant-mismatch, compared with the Match (*p* < 0.001), Irrelevant-mismatch (*p* < 0.001) and Conjunction-mismatch (*p* < 0.01); oscillatory theta power in Conjunction-mismatch was also stronger than Match (*p* < 0.01), no significant effect was found between Irrelevant-mismatch and Match (*p* = 1).

## Discussion

By using the delayed-matching task and its corresponding control task, the current study sought to examine the role of the frontal theta oscillation in detecting mismatched information during the comparison stage of VWM. We found that frontal distributed theta power was enhanced by both the relevant- and conjunction-mismatch in delayed matching task. However, these phenomena were not observed in the control task. The in-consistence of results between two tasks strongly indicates that frontal theta activity observed here reflected the VWM-related processing and played a constitutive role in detecting the mismatched information in VWM. For the functional significance of these oscillatory activities, someone may argue that they might be related to the response-related conflicts, as observed in the Flanker task (Hall et al., 2007; Cavanagh et al., 2009; Nigbur et al., 2012) and Stroop task (Hanslmayr et al., 2008). The combing of these studies showed that increased frontal theta power was consistently observed in the mismatch conditions of these tasks. More importantly, these oscillatory activities had the same changing trend as the behavioral conflicts of these tasks, indicating that frontal theta oscillations of these studies are conflict-related. However, this explanation is not compatible with the current findings. Our behavioral results showed that the RTs were significantly delayed under the relevant- and irrelevant-mismatch conditions in both tasks. While the differences in frontal theta power between conditions were only observed in delayed matching task and did not show the same changing trend as the behavioral conflicts. These results indicate that frontal theta activities observed in delayed matching task are not the neural index of external behavioral conflicts, but rather reflect the VWM-related mismatch detection processing.

In addition, we also examined whether the frontal theta power is related to the control process of VWM function. The behavioral results revealed that the irrelevant mismatch considerably obstructed the performance in the delayed matching task, suggesting that these irrelevant features were encoded into VWM. This result is consistent with previous studies (Magnussen et al., 1998; Hyun et al., 2009), and further supports the object-based encoding mechanisms in VWM (Shen et al., 2013). However, in delayed matching task we found that increased frontal theta power was observed in the relevant mismatch, rather than the irrelevant mismatch. These findings indicate that frontal theta activities observed in delayed matching task were top-down control related. This view was also supported by other data in the current study. In delayed matching task, we found that although the conjunction mismatch and the relevant mismatch elicited the same degree of behavioral conflicts. However, frontal theta power observed in the former was significantly lower than the latter. This suggests that when mismatch occurred simultaneously in both task relevant- and irrelevant-features, participants may take an active suppression strategy, rather than just ignoring it, to prevent the irrelevant mismatch from interfering with detecting the target-mismatched information. This explanation is consistent with the view that theta oscillation reflects a gating mechanism controlling task-relevant and suppressing the irrelevant information in WM processing (Raghavachari et al., 2001), and also further supports an existing theoretical proposal that oscillatory activities of the theta-band underlie the control process of working memory function (for review see Sauseng et al., 2010).

It has been proposed that the detection of mismatched information in the context of VWM task first triggers a rapid attentional shift to the spatial position of mismatched item (Pessoa and Ungerleider, 2004). This process was suggested to be mediated by the N2pc component (Hyun et al., 2009), which has a posterior distribution and indexes the deployment of spatial attention (Luck and Hillyard, 1994). After then, a top-down feedback is activated by the frontal network to initiate a more elaborate processing of the mismatched item (Pessoa and Ungerleider, 2004; Zhang et al., 2008). Notably, the time window (from 170 to 370 ms) of the frontal theta activity in current study was dramatically delayed in comparison with the peak latency of N2pc (around 200 ms in Hyun et al., 2009). This suggests that these oscillatory activities might reflect another processing stage after the rapid attentional shift (Hyun et al., 2009). Therefore, there may be a sequence-processing pattern in the mismatch detection process of VWM. It begins with the posterior bottom-up attention capture and then proceeds to the frontal control mechanism mediated by the theta-band oscillation.

Previous sensory recruitment hypothesis suggested that the posterior sensory regions involved in the initial encoding of the visual information are also considered as the primary storage sites for VWM (Serences et al., 2009). However, recent fMRI studies have pointed out that goal-related information (e.g., the target features in current study) is not stored in the sensory cortex but rather the higher frontal region (D’Esposito and Postle, 2015; Ester et al., 2015; Nee and D’Esposito, 2016). Through the integration of previous evidences and the current findings, a possible processing structure thus could be builded that underlies the mismatch detection processing in VWM: during the comparison stage of VWM, firstly, mismatched visual input triggers a bottom-up attentional capture to its position; then it is passed to the higher frontal region and is compared with the target representation to make a accurate judgment. This process is accomplished by the VWM-related control mechanism which mediated by the frontal theta oscillation. Notably, the above structure also indicates that the interaction between visual input and working memory contents in mismatch detection processing may be based on the neuronal mechanisms distinct from the biased competition model of visual selection, which suggests that top-down control signals from the target representation of working memory in favor of the item whose feature was pre-activated from working memory (Soto et al., 2008). The exploration of the differences in the brain mechanism between the two is undoubtedly a promising research.

## Conclusion

With elaborate experimental design, the current study clearly showed that frontal theta power supports detecting mismatched information during the comparison stage of VWM. Furthermore, we demonstrated that these oscillatory activities are related to the VWM-related control processing, rather than the general behavioral conflicts.

## Author Contributions

All authors designed the research, TL collected the data, TL and QL analyzed the data, and all authors interpreted the data and wrote the manuscript.

## Conflict of Interest Statement

The authors declare that the research was conducted in the absence of any commercial or financial relationships that could be construed as a potential conflict of interest.
